# 

**Published:** 1895-03

**Authors:** 


### Tri-State Meeting.

The Russell House, Detroit, has been selected as headquarters for the joint meeting of the State Societies of Michigan, Ohio and Indiana, which will occur June 18, 19 and 20. The clinics and scientific sessions will be held in the Dental Department of the Detroit College of Medicine and Surgery.

By order of Committee.

### EDITORIAL.

### Mississippi Valley Dental Society.

The fiftieth annual meeting of the Mississippi Valley Dental Society will be held in Cincinnati, on the 17th and 18th of April, 1895. An excellent programe has been prepared by the committee having the matter in charge. This will be sent out to the members and to many others who are and have been warm friends of this Pioneer Society in the past, within a few days. A history of the Society is being prepared by Dr. E. G. Betty which will doubtless be of great interest, especially to those who were identified with the Society in the earlier years of its existence.

It is confidently expected that there will be a large attendance, not only of the membership but of those, who have not been identified with it.

Come and learn what has been done by this, the Pioneer Society, and in addition to this, aid to make it a sucessful meeting.

### THE DENTAL REGISTER,

A MONTHLY MAGAZINE DEVOTED TO THE INTERESTS OF THE

**DENTAL PROFESSION.**

*Terms Reduced to* $2.00 *Per Year. Single Copies,* 25 *Cents.*

**EDITED BY PROF. J. TAFT, D.D.S.**

—AND—

**Published by SAM'L A. CROCKER & CO., Ohio Dental and Surgical Depot.**

The volume commences in January and closes in December.

The REGISTER being issued monthly is always fresh, therefore Indispensable to the practitioner who desires to keep posted up with the new inventions and improvements which are daily developed. Neither expense nor effort will be spared to give value and variety to its contents. Articles will be illustrated with well executed engravings whenever they will add to the interest or assist to explanation.

Its extensive circulation and frequency of issue make it one of the BEST DENTAL ADVERTISING MEDIUMS in the United States.

All subscriptions must begin with January or July.

Local or special notices, five lines or less, will be inserted for $3 each insertion; over five lines, 50 cts. per line.

All advertising bills payable in advance, or at furthest, after the issue of the first number containing the advertisement. All transient advertisements to be paid for in advance.

☞CONTRIBUTIONS SOLICITED.

Exclusively Editorial Communications should be addressed to the Editor.

The latest number of the REGISTER may be ordered with or without other goods at any time, and all letters should be addressed to

